# Association between healthy eating index-2015 scores and metabolic syndrome among Iranian women: a cross-sectional study

**DOI:** 10.1186/s12905-023-02876-1

**Published:** 2024-01-08

**Authors:** Mohammad-Reza Jowshan, Maryam Rafraf, Amir-Hossein Hashemi, Samaneh Hajjarzadeh, Mohammad Asghari-Jafarabadi, Somayyeh Asghari

**Affiliations:** 1 Department of Clinical Nutrition, School of Nutritional Sciences and Dietetics, Tehran University of Medical Sciences, No#44, Hojjatdoust St., Naderi St., Keshavarz Blvd, Tehran, 141556117 Iran; 2 Students’ Scientific Research Center, Tehran University of Medical Sciences, Tehran, Iran; 3 Nutrition Research Center, Department of Community Nutrition, Faculty of Nutrition and Food Science, Tabriz University of Medical Sciences, Tabriz, Iran; 4 Students’ Research Committee, Faculty of Nutrition and Food Science, Tabriz University of Medical Sciences, Tabriz, Iran; 5 Nutrition and Metabolic Diseases Research Center, Clinical Sciences Research Institute, Ahvaz Jundishapur University of Medical Sciences, Ahvaz, Iran; 6 Cabrini Research, Cabrini Health, 154 Wattletree Rd, Malvern, VIC 3144 Australia; 7 School of Public Health and Preventative Medicine, Faculty of Medicine, Nursing and Health Sciences, Monash University, Clayton, VIC 3800 Australia

**Keywords:** Metabolic syndrome, Diet, Healthy eating Index-2015, Women

## Abstract

**Background:**

Metabolic syndrome (MetS) is one of the leading public health issues in the world with a reported prevalence of nearly 25% in the past decades in Iran. The present research aimed to identify the association between the Healthy Eating Index (HEI) and MetS components among female teachers.

**Methods:**

In this cross-sectional study, 97 female teachers aged 31–57 years were enrolled from 2018 to 2019 in Qom, Iran. Usual dietary intakes were assessed using a validated 168-item Food Frequency Questionnaire (FFQ). HEI-2015 was calculated according to the consumption of whole fruits, vegetables, protein foods, beans, seafood, plant proteins, total and refined grain, dairy, fatty acid ratio, saturated fats, added sugars, and sodium. We also measured anthropometric and biochemical parameters. To evaluate the association between HEI-2015 and MetS, multivariate logistic regression was performed.

**Results:**

MetS was found in 59.8% of participants. Total HEI-2015 scores were significantly lower in participants with MetS compared to those without MetS (59.69 ± 8.98 vs. 64.21 ± 8.71, respectively; *p* = 0.02). Daily energy intake, weight, body mass index, waist circumference, systolic and diastolic blood pressure, serum triglyceride, and fasting blood sugar levels were higher in women with MetS (all *p* < 0.05). Higher HEI-2015 total scores (OR: 0.94; 95% CI: 0.89–0.99; *p* = 0.02) and scores of total vegetables (OR: 0.62; 95% CI: 0.42–0.91; *p* = 0.02), dark green vegetables and beans (OR: 0.62; 95% CI: 0.39–0.98; *p* = 0.04), fatty acid ratio (OR: 0.83; 95% CI: 0.68–0.99; *p* = 0.04), refined grain (OR: 0.86; 95% CI: 0.75–0.99; *p* = 0.04), and added sugars (OR: 0.44; 95% CI: 0.26–0.75; *p* = 0.002) were all associated with lower odds of having MetS.

**Conclusions:**

Higher HEI-2015 scores, particularly in total vegetables, dark green vegetables, beans, and fatty acid ratio, as well as refined grain and added sugars were found to be associated with reduced odds of having MetS among Iranian female teachers. However, further prospective studies are required to confirm this finding.

## Introduction

Metabolic syndrome (MetS) refers to the clustering of components characterized by abdominal obesity, hypertension, lipid disorders, and impaired glucose homeostasis [1]. Numerous definitions of MetS have been proposed by the World Health Organization (1999), the World Diabetes Federation (2006), and the American Heart Association (2005) [2]. However, the definition posed by the Adult Treatment Panel III (ATP III) is believed to be more appropriate for clinical applications [3]. According to the ATP III, the existence of three or more criteria indicates the presence of MetS. These criteria include elevated fasting blood sugar (FBS) to more than 100 mg/dl, serum triglyceride (TG) to more than 150 mg/dl, waist circumference (WC) to more than 88 cm in women and 102 cm in men, blood pressure (BP) to more than 130 mmHg for systolic or greater than 85 mmHg for diastolic, and decreased serum high-density lipoprotein cholesterol (HDL-C) levels to less than 50 mg/dl in women and less than 40 mg/dl in men [4]. MetS increases the risk of cardiovascular disease, type 2 diabetes mellitus, polycystic ovary syndrome, nonalcoholic fatty liver disease, cancers, and stroke [5]. It has been reported that about 10–25% of adults throughout the world [6] and 34.7% of the Iranian population suffer from MetS [7]. Furthermore, women are more prone to MetS than males in Middle Eastern countries [8].

Although the exact etiology of MetS remains unclear, it appears to be due to the interaction of genetic, metabolic, and environmental factors [9]. Lifestyle factors, like dietary intake, play an essential role in MetS pathophysiology [10]. Due to the fact that foods and macro-/micro-nutrients are rarely consumed in isolation, studying dietary patterns can help to examine the overall impact of the entire diet on MetS and its components, thereby providing a practical approach for dietary recommendations and MetS management [11–13]. In this regard, the Healthy Eating Index (HEI) has been considered to assess the diet quality in different societies with different food patterns, so that a higher HEI score indicates a higher diet quality [14]. It has been shown that the highest level of adherence to the HEI is significantly associated with a reduced risk of cardiovascular disease, cancer, type 2 diabetes mellitus, and all-cause mortality [15]. However, a multiethnic cohort study including over 89,185 participants failed to indicate any significant association between adherence to the HEI and the risk of type 2 diabetes; the authors attributed this issue to the ethnic differences and different consumption patterns of food components [16].

Since diet quality plays an important role in the prevention and management of chronic diseases, conducting studies in various populations is required for evaluating dietary intakes. A cross-sectional study among Iranian population showed that higher adherence to a high-quality diet based on Diet Quality Index-International (DQI-I) was associated with a lower chance of developing MetS in men, and not in the overall population [17]. However, another study did not show a significant association between adherence to the Main Meal Quality Index (MMQI) and MetS and its components among Iranian men and women [18].

There is limited research to determine diet quality based on HEI and the MetS risk among Iranian populations. Only one study reported that higher adherence to the HEI-2010 total score reduced the risk of MetS by 28% among Iranian adult women [19]; however, HEI-2010 component scores were not evaluated with the risk of MetS. Considering the higher prevalence of MetS among women, it is essential to evaluate the diet quality of women to make healthy diet recommendations for MetS management. Accordingly, the present study was conducted from 2018 to 2019 to investigate the relationship between HEI-2015 scores and MetS among female teachers in Qom, Iran.

## Methods

### Participants

This cross-sectional study included non-menopausal female teachers aged 30–55 years to minimize the confounding effect of hormonal changes in menopause on the association between HEI-2015 scores and MetS [20]. The sample size was determined based on the information extracted from the study by Shahvazi S et al. [21]. Considering a 95% confidence level, 80% statistical power, an odds ratio (OR) of 0.48 for MetS in the highest tertile compared with the lowest tertile of HEI-2010 score, and a 42% prevalence rate of MetS among Iranian women [19], the sample size was calculated as 97 subjects using the G*Power (version 3.1) software. To minimize selection bias, multistage cluster sampling was performed. In the first stage, the regions were selected based on the classification of school districts. In the second stage, a proportional number of women’s schools were randomly selected in each district. Finally, in the third stage, the individuals were selected by convenience sampling in each school. After confirming the inclusion and exclusion criteria, all individuals voluntarily participated in the study. Pregnant and lactating women were not included in this study. Moreover, smoking, following a special diet, being a professional athlete, and taking medications such as systemic steroids, nonsteroidal anti-inflammatory drugs (NSAIDs), and antipsychotics were considered as the exclusion criteria.

### Data collection

We collected the general information of participants, including age, marital status, medical history, as well as medication and dietary supplement using a questionnaire. Anthropometric data, including height, weight, body mass index (BMI), and WC were obtained under standardized conditions. Height was measured without shoes in an upright position using a fixed non-stretchable tape with a precision of 0.5 cm. Weight was measured in light clothing by a Seca scale to the nearest 0.1 kg. BMI was calculated as weight (kg) divided by squared height (m^2^). Waist and hip circumferences were measured at the narrowest level and largest part, respectively, with a non-stretchable tape and recorded to the nearest 0.1 cm. Clinical and biochemical examinations included assessment of blood pressure, HDL-C, TG, and fasting blood sugar (FBS). Systolic blood pressure (SBP) and diastolic blood pressure (DBP) were measured twice with a sphygmomanometer in the right arm after 10 min of rest, and the mean of the two values was used in the analysis. Venous blood samples were drawn after an overnight fasting, and serum samples were stored at 70 °C until analysis. Serum TG and HDL-C concentrations were measured by enzymatic techniques and FBS concentration was determined by the glucose oxidase method using commercial kits (Pars Azmoon Inc. Iran).

Dietary intake over the past year was assessed semi-quantitatively using the Food Frequency Questionnaire (FFQ). The FFQ used in this study consists of a list of 168 foods and food items along with a standard size of each food item, designed according to the Willett method. Because an FFQ is composed of a pre-specified food list, any single FFQ may not reflect the eating patterns of a given population. Since FFQs are prone to measurement error, several studies have been conducted among Iranian population, all confirming the validity and reliability for food group intake, nutrients, and dietary patterns [22–24]. After completing the FFQ, the consumed foods were coded according to the instructions of Nutrition 4 software to determine energy and nutrients.

The HEI-2015 is the latest version of this index designed in 2015 under the 2015–2020 dietary guidelines for Americans [14]. In this method, the scores are assigned based on 13 food groups with a total maximum score of 100 [25]. The nine adequacy components of HEI-2015 contain total fruits, whole fruits, total vegetables, greens and beans, whole grains, dairy, total protein foods, seafood and plant proteins, and fatty acids. It also consists of four moderation components, including refined grains, sodium, added sugars, and saturated fats. Each of the components is scored on a density basis out of 1,000 calories except fatty acids, which is the ratio of unsaturated to saturated fatty acids. For adequacy components, the minimum and maximum intakes can range from 0 to 5, respectively [14, 25]. However, regarding dairy, whole grains, and fatty acids, the maximum score is 10. Considering the moderation components, the intakes fall within the range of 0 to 10 (the lowest intake gets 10 and the highest intake gets 0) (Table 1). HEI-2015 scores were obtained from the sum of 13 components from each participant (10). Also, the HEI score < 50 was considered as poor, 50 to 80 as needs improvement, and > 80 as good [14, 25].

**Table 1:** HEI–2015^*^ components & scoring standards

Component	Maximum points	Standard for maximum score	Standard for minimum score of zero
**Adequacy:**			
Total Fruits^†^	5	≥ 0.8 cup equiv. per 1,000 kcal	No Fruits
Whole Fruits^‡^	5	≥ 0.4 cup equiv. per 1,000 kcal	No Whole Fruits
Total Vegetables^§^	5	≥ 1.1 cup equiv. per 1,000 kcal	No Vegetables
Greens and Beans^§^	5	≥ 0.2 cup equiv. per 1,000 kcal	No Dark Green Vegetables or Legumes
Whole Grains	10	≥ 1.5 oz equiv. per 1,000 kcal	No Whole Grains
Dairy^||^	10	≥ 1.3 cup equiv. per 1,000 kcal	No Dairy
Total Protein Foods^¶^	5	≥ 2.5 oz equiv. per 1,000 kcal	No Protein Foods
Seafood and Plant Proteins^¶**^	5	≥ 0.8 oz equiv. per 1,000 kcal	No Seafood or Plant Proteins
Fatty Acids^††^	10	(PUFAs + MUFAs)/SFAs ≥ 2.5	(PUFAs + MUFAs)/SFAs ≤ 1.2
**Moderation**:			
Refined Grains	10	≤ 1.8 oz equiv. per 1,000 kcal	≥ 4.3 oz equiv. per 1,000 kcal
Sodium	10	≤ 1.1 gram per 1,000 kcal	≥ 2.0 g per 1,000 kcal
Added Sugars	10	≤ 6.5% of energy	≥ 26% of energy
Saturated Fats	10	≤ 8% of energy	≥ 16% of energy

HEI-2015, Healthy Eating Index-2015

*Intakes between the minimum and maximum standards are scored proportionately. The total HEI score is the sum of the adequacy components (i.e. foods to eat more of for good health) and moderation components (i.e. foods to limit for good health)

†Includes 100% fruit juice

‡Includes all forms except juice

§Includes legumes (beans and peas)

||Includes all milk products, such as fluid milk, yogurt, and cheese, and fortified soy beverages

¶Includes legumes (beans and peas)

**Includes seafood, nuts, seeds, soy products (other than beverages), and legumes (beans and peas)

††Ratio of poly- and monounsaturated fatty acids (PUFAs and MUFAs) to saturated fatty acids (SFAs)

Physical activity was assessed using the Iranian version of the International Physical Activity Questionnaires (IPAQ) and metabolic equivalent hours per week (MET-h per week) and then categorized as “light”, “moderate”, and “heavy” activity [26]. All measurements and interviews were conducted by a trained nutritionist.

### Statistical analysis

The normal distribution of data was checked by Kolmogorov–Smirnov test. Regarding quantitative data, normal data were presented as mean ± standard deviation and non-normal data as median (interquartile range). Meanwhile, qualitative data were presented as frequency (percentage). To determine the association between HEI total and component scores with MetS, multivariable logistic regression analysis was used in different models. First, the confounding effect of age and BMI was controlled (Model 2). Further statistical control was performed for daily energy intake and physical activity level (Model 3). All statistical analyses were performed using the SPSS (Chicago IL, USA; version 18) and *P* < 0.05 was considered significant.

## Results

### Subjects’ characteristics

This study was performed on 97 female teachers with a mean age of 43.87 ± 7.14 years. The general and metabolic characteristics of participants are shown in Table 2. Women with MetS (n = 58) had significantly higher daily energy intake, weight, BMI, WC, SBP, DBP, serum TG, and FBS levels compared to the women without MetS (n = 39). However, physical activity level and serum HDL-C had no significant differences between the two groups (Table 2).

**Table 2:** Characteristics of the study participants

Variables	Total N = 97		Women with MetS N = 58		Women without MetS N = 39		***P***-value
	Mean	SD	Mean	SD	Mean	SD	
**Age**	44	13	47	14	41	12	**0.001**
**Daily energy intake (kcal)**	1797	520	2067	439	1737	303	**< 0.001**
**Weight (kg)**	64.5	9.1	69.17	8.26	62.5	9	**< 0.001**
**Body mass index (kg/m** ^**2**^ **)**	24.69	4.06	25.91	4.17	23.15	3.79	**< 0.001**
	**N (%)**		**N (%)**		**N (%)**		
**Body mass index category**							
Low weight (< 18.5)	0		0		0		**< 0.001**
Normal (18.5 to 24.99)	52 (53.6%)		11 (28.2%)		41 (70.7%)		
Overweight (25 to 29.99)	36 (37.1%)		20 (51.3%)		16 (27.6%)		
Obese (≥ 30)	9 (9.36%)		8 (20.5%)		1 (1.7%)		
**Waist circumference (cm)**							
≥ 88	41 (42.3%)		28 (71.8%)		13 (22.4%)		**< 0.001**
< 88	56 (57.7%)		11 (28.2%)		45 (77.6%)		
**Systolic blood pressure (mmHg)**							
≥ 130	24 (24.7%)		16 (41%)		8 (13.8%)		**0.002**
< 130	73 (75.3%)		23 (59%)		50 (86.2%)		
**Diastolic blood pressure (mmHg)**							
≥ 85	28 (28.9%)		20 (51.3%)		8 (13.8%)		**< 0.001**
< 85	69 (71.1%)		19 (48.7%)		50 (86.2%)		
**Fasting blood sugar (mg/dL)**							
≥ 100	17 (17.5%)		11 (28.2%)		6 (10.3%)		**0.02**
< 100	80 (82.5%)		28 (71.8%)		52 (89.7%)		
**Triglyceride (mg/dL)**							
> 150	33 (34%)		28 (71.8%)		5 (8.6%)		**< 0.001**
≤ 150	64 (66%)		11 (28.2%)		53 (91.4%)		
**HDL-C (mg/dL)**							
< 50	79 (81.4%)		33 (84.6%)		46 (79.3%)		0.51
≥ 50	18 (18.6%)		6 (15.4%)		12 (20.7%)		
**Physical activity category (MET-h per week)**							
light (MET < 600)	8 (8.2%)		4 (10.3%)		4 (6.9%)		0.70
moderate (600 ≤ MET < 3000)	82 (84.5%)		33 (84.6%)		49 (84.5%)		
heavy (MET ≥ 3000)	7(7.2%)		2 (5.1%)		5 (8.6%)		

MetS, metabolic syndrome; HDL-C, high-density lipoprotein cholesterol; MET, metabolic equivalent of task

Data are reported as mean ± standard deviation or frequency (percent)

*P*-values based on independent sample t-test or Pearson chi-square test

### Association of HEI components with metabolic markers

Table 3 presents the HEI components’ scores along with the total score of HEI for women with and without MetS.

**Table 3:** Comparing the HEI scores of women with and without metabolic syndrome

HEI-2015 Total/Component Score	Total (N = 97)		Women with MetS (N = 58)		Women without MetS (N = 39)		***P***-values*
	Mean/ Median	SD/IQR	Mean/ Median	SD/IQR	Mean/ Median	SD/IQR	
Total fruit	3.11	1.55	3.34^£^	1.08^£^	3.09	1.15	0.28
Whole fruit	5	0.36	5	0.13	5	0.77	0.25
Total vegetables	4.25	1.79	3.70	2.17	4.82	1.64	0.01
Dark green vegetables and beans	5	0.97	5	1.40	5	0.68	0.06
Whole grain	10	3	9.70	3.90	10	1.81	0.11
Dairy products	5.17	2.39	5.07^£^	1.70^£^	5.57	2.70	0.14
Total protein foods	2.78	1.4	2.49	1.01	3.11^£^	0.96^£^	0.02
Seafood and plant proteins	4.14	2.03	3.85	2.50	4.19	1.54	0.13
Fatty acid ratio	4.33	4.89	3.28	4.53	5.09^£^	2.74^£^	0.11
Refined grain	6.35	6.97	5.17	6.98	7.12	7.39	0.09
Added sugars	10	1.01	9.84^£^	1.88^£^	10	0.1	0.002
Sodium	0		0		0		-
Saturated fats	5.55	3.74	6.08^£^	2.38^£^	5.38^£^	2.76^£^	0.20
**Total HEI score**	62.40^£^	9.05^£^	59.69^£^	8.98^£^	64.21^£^	8.71^£^	0.02

HEI-2015, Healthy Eating Index-2015; SD, Standard Deviation; IQR, Interquartile Range

Normally distributed variables were reported using mean (SD) and non-normally distributed variables were reported using median (IQR)

^£^ Mean (SD)

* *P*-values based on independent sample t-test (for normally distributed variables) and Mann-Whitney test (for non-normally distributed variables)

According to the results, while whole grain and added sugars had the highest score in women with MetS, sodium, total protein foods, and total fruit had the lowest scores in them. Also, the mean scores of “total vegetables” (*p* = 0.01), “total protein foods” (*p* = 0.02), and “added sugars” (*p* = 0.002) were significantly lower in women with MetS. There were no significant differences between the two groups regarding the scores of other HEI components. Moreover, the mean (± standard deviation) of HEI total score in women with MetS was significantly lower than that of women without MetS (59.69 ± 8.98 vs. 64.21 ± 8.71, respectively; *p* = 0.02).

The results of multivariable-adjusted ORs for MetS risk across HEI components’ scores, as well as HEI total score, are outlined in Table 4.

**Table 4:** The multiple regression model to assess the relation of metabolic syndrome risk with HEI components’ scores

HEI components	Model 1	Model 2	Model 3	HEI components	Model 1	Model 2	Model 3
**Total fruit**				**Seafood and plant proteins**			
OR (95%CI)	1.23 (0.85–1.77)	1.04 (0.68–1.60)	1.08 (0.69–1.66)	OR (95%CI)	0.72 (0.49–1.05)	0.78 (0.51–1.20)	0.78 (0.50–1.22)
*P*-value	0.28	0.86	0.74	*P*-value	0.09	0.26	0.28
**Whole fruit**				**Fatty acid ratio**			
OR (95%CI)	1.51 (0.82–2.76)	1.24 (0.63–2.43)	1.33 (0.68–2.60)	OR (95%CI)	0.90 (0.77–1.04)	0.85 (0.72–1.01)	**0.83 (0.68–0.99)**
*P*-value	0.19	0.54	0.41	*P*-value	0.15	0.07	**0.04**
**Total vegetables**				**Refined grain**			
OR (95%CI)	**0.62 (0.42–0.91)**	0.65 (0.41–1.03)	0.65 (0.41–1.04)	OR (95%CI)	0.92 (0.83–1.03)	1.08 (0.78–1.02)	**0.86 (0.75–0.99)**
*P*-value	**0.02**	0.07	0.07	*P*-value	0.17	0.12	**0.04**
**Dark green vegetables and beans**				**Added sugars**			
OR (95%CI)	**0.62 (0.39–0.98)**	0.87 (0.49–1.49)	0.87 (0.49–1.55)	OR (95%CI)	**0.44 (0.26–0.75)**	**0.48 (0.26–0.88)**	**0.48 (0.26–0.90)**
*P*-value	**0.04**	0.58	0.64	*P*-value	**0.002**	**0.02**	**0.02**
**Whole grain**				**Saturated fats**			
OR (95%CI)	0.90 (0.76–1.06)	0.87 (0.72–1.05)	0.87 (0.71–1.06)	OR (95%CI)	1.11 (0.95–1.30)	1.06 (0.88–1.28)	1.06 (0.87–1.29)
*P*-value	0.20	0.16	0.15	*P*-value	0.20	0.55	0.57
**Dairy products**				**Total HEI scores**			
OR (95%CI)	0.87 (0.71–1.06)	1.01 (0.79–1.30)	1.05 (0.81–1.35)	OR (95%CI)	**0.94 (0.89–0.99)**	**0.94 (0.88–0.99)**	**0.93 (0.88–0.99)**
*P*-value	0.16	0.92	0.71	*P*-value	**0.02**	**0.03**	**0.02**
**Total protein foods**				**Model 1: Crude model.** **Model 2: Adjusted for age and BMI.** **Model 3: Model 2 + Adjusted for daily energy intake and physical activity level.** **P- values based on logistic regression**			
OR (95%CI)	0.65 (0.42–1.003)	0.62 (0.36–1.05)	0.59 (0.35–1.02)				
*P*-value	0.05	0.07	0.06				

The scores of total vegetables (OR: 0.62; 95% CI: 0.42–0.91; *p* = 0.02), as well as dark green vegetables and beans (OR: 0.62; 95% CI: 0.39–0.98; *p* = 0.04) were associated with lower odds of having MetS in the crude model. After adjusting for age, BMI, daily energy intake, and physical activity level, higher scores of fatty acid ratio (OR: 0.83; 95% CI: 0.68–0.99; *p* = 0.04) and refined grain (OR: 0.86; 95% CI: 0.75–0.99; *p* = 0.04) were associated with lower odds of having MetS. Also, the score of added sugars and the HEI total score were associated with lower odds of MetS in both crude and adjusted models (Table 4).

## Discussion

MetS, characterized by abdominal obesity, hypertension, lipid disorders, and impaired glucose homeostasis, is associated with the risk of cardiovascular disease, type 2 diabetes, polycystic ovary syndrome, non-alcoholic fatty liver disease, cancer, and stroke [5]. Diet, as an efficient environmental factor, has an important role in the management of MetS [9]. One of the issues considered in recent years is using methods to evaluate the dietary patterns and the quality of the diet consumed by individuals. According to some previous studies, higher quality of the diet and conformity to the HEI have been associated with a lower risk of developing MetS among women [27]. There are numerous studies investigating the association between HEI and various chronic diseases worldwide. Although dietary patterns and quality of the diet have been extensively studied, the optimal diet for MetS has not been known yet [28, 29].

In the present research, there was an inverse association between the HEI-2015 total score and the odds of having MetS. Similarly, in a previous study, an inverse association between MetS and HEI was reported in 226 elderly [30]. In another study conducted on 4,450 American adolescents, MetS prevalence decreased by increasing the score of HEI [31]. Tardivo et al. showed an association between HEI and metabolic risk in 173 postmenopausal women [32]. The mean of the HEI score in the study by Tardivo et al. was almost similar to that of our study. However, Heydari-Araghi et al. found no significant difference regarding the HEI-2010 score between individuals with and without MetS [33]. The mean HEI score in the study by Heydari-Araghi et al. was 73.64, which was higher than our study; this may affect the results. These contradictions in the current evidence are probably because of the heterogeneity in study designs, measured outcomes, sample sizes, specific food culture and habits, study population socio-demographic characteristics, and the used assessment tools.

We also found that a higher intake of total vegetables, as well as dark green vegetables and beans were associated with lower odds of having MetS. In addition, a higher ratio of poly- and mono-unsaturated fatty acids to saturated fatty acids in the diet was inversely associated with MetS. Lower intake of refined grain and added sugars were also associated with a lower risk of MetS. Limited studies have determined the association between HEI-2015 component scores and MetS among women. Azadbakht et al. conducted a cross-sectional study, indicating an inverse relationship between fruits and vegetable consumption and MetS among women in Tehran [34]. Hooshmand et al. demonstrated that higher scores of modified HEI components such as fruits, salty snacks, and fast foods were associated with a decreased risk of MetS in 424 healthy individuals aged 6–18 years [35]. Tardivo et al. also showed that the association between poor quality of the diet and metabolic risk in postmenopausal women was related with low whole-grain intake and high saturated fat consumption [32]. Higher intake of saturated fatty acids had a potential role in insulin resistance status and could contribute to MetS development [36]. Contrary to the study by Esmaillzadeh et al. [37], Lutsey et al. [38] demonstrated no significant relationship between fruits and vegetable intake with MetS incidence. Although Heydari-Araghi et al. found no significant difference in HEI-2010 score between individuals with and without MetS, they showed that the whole fruit score was lower in subjects with MetS compared to healthy subjects. However, they did not find any significant differences in total vegetables, dark green, orange vegetables, and legume scores between the two groups [33]. It is assumed that the association of fruits, vegetables, and whole grains with MetS is mediated through their high content of fiber, phytochemicals, and antioxidants [31]. It has been shown that each 3 g/1000 kcal increase in fiber intake could decrease the risk of MetS by 34% [39]. Potential mechanisms have been proposed to explain this relationship. Fruits, vegetables, and whole grains contain fiber, phytochemicals, and antioxidants, which have been shown to reduce blood sugar levels and improve insulin resistance [3]. Phytochemicals have antioxidant and anti-inflammatory properties that can reduce inflammation in the body. Antioxidants can also help protect cells from damage caused by free radicals [40].

According to the mentioned studies, the relationship between HEI components and MetS may be conflicting. There may be several contradictory factors that could affect the results, including the cross-sectional nature of most of the studies, the application of the FFQ questionnaire which is memory-based, race and ethnicity, genetic differences, and the way of food processing and cooking like using fried vegetables versus steamed or raw consumed vegetables. Different cooking methods alter the bioavailability of nutrients and their structure [41, 42].

The major strengths of this study include data analysis after modifying the potential confounders, using valid questionnaires, and in-person interviews by trained nutritionists. However, this research had several limitations, including the probable selection bias in the sampling process, the impossibility of determining causality due to the cross-sectional nature of the study, and inherent FFQ limitation which is memory-based and may cause recall bias. Prior knowledge regarding the MetS condition might influence the subjects’ lifestyle and diet, which could result in bias. In addition, some unknown or unmeasured confounding variables such as socioeconomic and cultural differences might affect these findings. It should also be noted that our findings do not apply to all female populations or males. Further studies are required to investigate the associations between diet quality and MetS risk in other populations.

## Conclusion

According to the results of this study, higher HEI-2015 scores, particularly in total vegetables, dark green vegetables, beans, fatty acid ratio, refined grain, and added sugars were found to be associated with reduced odds of having MetS among Iranian female teachers. Thus, the HEI-2015 score might be an appropriate predictor for the relationship between diet quality and MetS among Iranian women based on the associations between the scores of these specific components with the odds of having MetS. To confirm these findings, further prospective cohort studies are required to evaluate the causal effects between HEI-2015 scores and MetS risk over time.

## Data Availability

On reasonable request, the corresponding author will provide the datasets used and analyzed during the current work.

## Abbreviations

HEI: Healthy Eating Index
FFQ: Food Frequency Questionnaire
BMI: Body Mass Index
MetS: Metabolic syndrome
TG: Triglyceride
FBS: Fasting Blood Sugar
AHA: American Heart Association
WHO: World Health Organization
ATP III: Adult Treatment Panel III
WC: Waist Circumference
HDL-C: High-Density Lipoprotein Cholesterol
SBP: Systolic Blood Pressure
DBP: Diastolic Blood Pressure
NSAIDs: Nonsteroidal Anti-Inflammatory Drugs
IPAQ: International Physical Activity Questionnaires
MET-h per week: Metabolic Equivalent Hours per week
SE: Standard Error
ORs: Adjusted Odds Ratios

## References

[1] Sherling DH, Perumareddi P, Hennekens CH (2017). Metabolic syndrome: clinical and policy implications of the new silent killer. J Cardiovasc Pharmacol Therap.

[2] Ambroselli D, Masciulli F, Romano E, Catanzaro G, Besharat ZM, Massari MC, Ferretti E, Migliaccio S, Izzo L, Ritieni A (2023). New advances in metabolic syndrome, from Prevention to Treatment: the role of Diet and Food. Nutrients.

[3] Huang PL (2009). A comprehensive definition for metabolic syndrome. Dis Models Mech.

[4] Heyn PC, Tagawa A, Pan Z, Thomas S, Carollo JJ (2019). Prevalence of metabolic syndrome and Cardiovascular Disease risk factors in adults with cerebral palsy. Dev Med Child Neurol.

[5] Mendrick DL, Diehl AM, Topor LS, Dietert RR, Will Y, La Merrill MA, Bouret S, Varma V, Hastings KL, Schug TT (2018). Metabolic syndrome and associated Diseases: from the bench to the clinic. Toxicol Sci.

[6] Akbaraly TN, Singh-Manoux A, Tabak AG, Jokela M, Virtanen M, Ferrie JE, Marmot MG, Shipley MJ, Kivimaki M (2010). Overall diet history and reversibility of the metabolic syndrome over 5 years: the Whitehall II prospective cohort study. Diabetes Care.

[7] Sayehmiri F (2014). Metabolic syndrome prevalence in Iran: a systematic review and meta-analysis. J Kermanshah Univ Med.

[8] Saklayen MG (2018). The global epidemic of the metabolic syndrome. Curr Hypertens Rep.

[9] Pitsavos C, Panagiotakos D, Weinem M, Stefanadis C (2006). Diet, exercise and the metabolic syndrome. Rev Diabet Stud.

[10] Djousse L, Padilla H, Nelson T, Gaziano J, Mukamal K (2010). Diet and metabolic syndrome. *Endocrine, metabolic & Immune disorders-drug targets (formerly current drug targets-Immune*. Endocr Metabolic Disorders).

[11] Esmaillzadeh A, Mirmiran P, Azizi F (2005). Whole-grain consumption and the metabolic syndrome: a favorable association in tehranian adults. Eur J Clin Nutr.

[12] Saneei P, Fallahi E, Barak F, Ghasemifard N, Keshteli AH, Yazdannik AR, Esmaillzadeh A (2015). Adherence to the DASH diet and prevalence of the metabolic syndrome among Iranian women. Eur J Nutr.

[13] Mullie P, Clarys P, Hulens M, Vansant G (2010). Dietary patterns and socioeconomic position. Eur J Clin Nutr.

[14] Krebs-Smith SM, Pannucci TE, Subar AF, Kirkpatrick SI, Lerman JL, Tooze JA, Wilson MM, Reedy J (2018). Update of the healthy eating index: HEI-2015. J Acad Nutr Dietetics.

[15] Schwingshackl L, Hoffmann G (2015). Diet quality as assessed by the healthy eating Index, the alternate healthy eating Index, the Dietary approaches to stop Hypertension score, and health outcomes: a systematic review and meta-analysis of cohort studies. J Acad Nutr Dietetics.

[16] Jacobs S, Harmon BE, Boushey CJ, Morimoto Y, Wilkens LR, Le Marchand L, Kröger J, Schulze MB, Kolonel LN, Maskarinec G (2015). A priori-defined diet quality indexes and risk of type 2 Diabetes: the multiethnic cohort. Diabetologia.

[17] Ghalandari H, Askarpour M, Nouri M, Safarpour AR, Fattahi MR, Akbarzadeh M. Quality of Diet and Odds of Metabolic Syndrome in Iranian Adults: Baseline Results from the PERSIAN Kavar Cohort Study (PKCS). *Nutrition, Metabolism and Cardiovascular Diseases* 2023. 10.1016/j.numecd.2023.04.017 37414660

[18] Mirrafiei A, Hasanzadeh M, Sheikhhossein F, Majdi¹ M, Djafarian K, Shab-Bidar S (2023). Association of main meal quality index with the odds of metabolic syndrome in Iranian adults: a cross-sectional study. BMC Nutr.

[19] Saraf-Bank S, Haghighatdoost F, Esmaillzadeh A, Larijani B, Azadbakht L (2017). Adherence to healthy eating Index-2010 is inversely associated with metabolic syndrome and its features among Iranian adult women. Eur J Clin Nutr.

[20] El Khoudary SR, Aggarwal B, Beckie TM, Hodis HN, Johnson AE, Langer RD, Limacher MC, Manson JE, Stefanick ML, Allison MA (2020). Menopause transition and Cardiovascular Disease risk: implications for timing of early prevention: a scientific statement from the American Heart Association. Circulation.

[21] Shahvazi S, Barak F, Heydari M, Saneie P (2015). Association between Healthy Eating Index and metabolic syndrome in women: a Cross Sectional Study. J Ilam Univ Med Sci.

[22] Esfahani FH, Asghari G, Mirmiran P, Azizi F (2010). Reproducibility and relative validity of food group intake in a food frequency questionnaire developed for the Tehran lipid and glucose study. J Epidemiol.

[23] Mirmiran P, Esfahani FH, Mehrabi Y, Hedayati M, Azizi F (2010). Reliability and relative validity of an FFQ for nutrients in the Tehran lipid and glucose study. Public Health Nutr.

[24] Asghari G, Rezazadeh A, Hosseini-Esfahani F, Mehrabi Y, Mirmiran P, Azizi F (2012). Reliability, comparative validity and stability of dietary patterns derived from an FFQ in the Tehran lipid and glucose study. Br J Nutr.

[25] Reedy J, Lerman JL, Krebs-Smith SM, Kirkpatrick SI, Pannucci TE, Wilson MM, Subar AF, Kahle LL, Tooze JA (2018). Evaluation of the healthy eating index-2015. J Acad Nutr Dietetics.

[26] Moghaddam MB, Aghdam FB, Jafarabadi MA, Allahverdipour H, Nikookheslat SD, Safarpour S (2012). The Iranian version of International Physical Activity Questionnaire (IPAQ) in Iran: content and construct validity, factor structure, internal consistency and stability. World Appl Sci J.

[27] Camhi SM, Evans EW, Hayman LL, Lichtenstein AH, Must A (2015). Healthy eating index and metabolically healthy obesity in US adolescents and adults. Prev Med.

[28] Sonnenberg L, Pencina M, Kimokoti R, Quatromoni P, Nam BH, D’agostino R, Meigs JB, Ordovas J, Cobain M, Millen B (2005). Dietary patterns and the metabolic syndrome in obese and non-obese Framingham women. Obes Res.

[29] Esmaillzadeh A, Kimiagar M, Mehrabi Y, Azadbakht L, Hu FB, Willett WC (2007). Dietary patterns, insulin resistance, and prevalence of the metabolic syndrome in women. Am J Clin Nutr.

[30] Kord Varkaneh H, Rrahmani J, Shab-Bidar S (2018). Association of adherence to Alternative Healthy Eating Index with the metabolic syndrome in Tehranian elderly. Razi J Med Sci.

[31] Pan Y, Pratt CA (2008). Metabolic syndrome and its association with diet and physical activity in US adolescents. J Am Diet Assoc.

[32] Tardivo AP, Nahas-Neto J, Nahas EA, Maesta N, Rodrigues MA, Orsatti FL (2010). Associations between healthy eating patterns and indicators of metabolic risk in postmenopausal women. Nutr J.

[33] Heydari AM, Mozaffari KH, Jafaraian K, Esteghamati A, Meysami A, Montazeripour S. Comparison of healthy eating index between individuals with and without metabolic syndrome. 2012.

[34] Esmaillzadeh A, Azadbakht L (2007). The effects of fruit and vegetable intakes on C-reactive protein and the metabolic syndrome among women. Iran J Diabetes Metabolism.

[35] Hooshmand F, Asghari G, Yuzbashian E, Mahdavi M, Mirmiran P, Azizi F (2018). Modified healthy eating index and incidence of metabolic syndrome in children and adolescents: Tehran lipid and glucose study. J Pediatr.

[36] Esmaillzadeh A, Azadbakht L (2008). Consumption of hydrogenated versus nonhydrogenated vegetable oils and risk of insulin resistance and the metabolic syndrome among Iranian adult women. Diabetes Care.

[37] Esmaillzadeh A, Kimiagar M, Mehrabi Y, Azadbakht L, Hu FB, Willett WC (2006). Fruit and vegetable intakes, C-reactive protein, and the metabolic syndrome. Am J Clin Nutr.

[38] Lutsey PL, Steffen LM, Stevens J. Dietary intake and development of the metabolic syndrome: The atherosclerosis risk in communities study. In: *Circulation: 2007*:Lippincott Williams & Wilkins 530 Walnut St, Philadelphia, PA 19106 – 3621 USA; 2007:E220-E220. 10.1161/CIRCULATIONAHA.107.716159 18212291

[39] Pereira MA, Jacobs DR, Van Horn L, Slattery ML, Kartashov AI, Ludwig DS (2002). Dairy consumption, obesity, and the insulin resistance syndrome in young adults: the CARDIA Study. JAMA.

[40] Feinkohl I, Janke J, Hadzidiakos D, Slooter A, Winterer G, Spies C, Pischon T (2019). Associations of the metabolic syndrome and its components with cognitive impairment in older adults. BMC Geriatr.

[41] Moreno DA, López-Berenguer C, García‐Viguera C (2007). Effects of stir‐fry cooking with different edible oils on the phytochemical composition of broccoli. J Food Sci.

[42] Yuan G-f, Sun B, Yuan J, Wang Q-m (2009). Effects of different cooking methods on health-promoting compounds of broccoli. J Zhejiang Univ Sci B.

